# The ranging of amino acids substitution matrices of various types in accordance with the alignment accuracy criterion

**DOI:** 10.1186/s12859-020-03616-0

**Published:** 2020-09-14

**Authors:** Valery Polyanovsky, Alexander Lifanov, Natalia Esipova, Vladimir Tumanyan

**Affiliations:** Engelhardt Institute of Molecular Biology of Russian Academy of Sciences, Vavilova 32, 119991 Moscow, Russia

**Keywords:** Evolutionary distance, Divergent evolution, Sequence alignment, Substitution matrix, Penalcty function

## Abstract

**Background:**

The alignment of character sequences is important in bioinformatics. The quality of this procedure is determined by the substitution matrix and parameters of the insertion-deletion penalty function. These matrices are derived from sequence alignment and thus reflect the evolutionary process. Currently, in addition to evolutionary matrices, a large number of different background matrices have been obtained. To make an optimal choice of the substitution matrix and the penalty parameters, we conducted a numerical experiment using a representative sample of existing matrices of various types and origins.

**Results:**

We tested both the classical evolutionary matrix series (PAM, Blosum, VTML, Pfasum); structural alignment based matrices, contact energy matrix, and matrix based on the properties of the genetic code. This study presents results for two test set types: first, we simulated sequences that reflect the divergent evolution; second, we performed tests on Balibase sequences. In both cases, we obtained the dependences of the alignment quality (*Accuracy*, *Confidence*) on the evolutionary distance between sequences and the evolutionary distance to which the substitution matrices correspond. Optimization of a combination of matrices and the penalty parameters was carried out for local and global alignment on the values of penalty function parameters.

Consequently, we found that the best alignment quality is achieved with matrices corresponding to the largest evolutionary distance. These matrices prove to be universal, i.e. suitable for aligning sequences separated by both large and small evolutionary distances. We analysed the correspondence of the correlation coefficients of matrices to the alignment quality. It was found that matrices showing high quality alignment have an above average correlation value, but the converse is not true.

**Conclusions:**

This study showed that the best alignment quality is achieved with evolutionary matrices designed for long distances: Gonnet, VTML250, PAM250, MIQS, and Pfasum050. The same property is inherent in matrices not only of evolutionary origin, but also of another background corresponding to a large evolutionary distance. Therefore, matrices based on structural data show alignment quality close enough to its value for evolutionary matrices. This agrees with the idea that the spatial structure is more conservative than the protein sequence.

## Background

Alignment is the most common bioinformatics procedure.A natural quality criterion for the alignment procedure is the reproducibility of true alignment, i.e., the restoration of true events at the level of substitutions and insertion-deletions of amino acids in a symbolic sequence. To be precise, as applied to the scheme of divergent evolution, this is a comparison of positions in two mutant sequences originating from the same position of a common ancestor. Thus, the natural criterion for the matrix performance should be the effectiveness of the alignment procedure using this matrix.

### Classification of matrices according to the principle of obtaining

Existing amino acid substitution matrices can be divided into groups in accordance with the principles by which they were derived. The most representative group in terms of the number of matrices and applicability for the alignment procedure should include matrices of evolutionary nature, i.e. matrices obtained by comparing sequences. The most famous representatives of this group include matrices of universal application. These are the matrices series of PAM [1], Blosum [2], as well as VTML [3] and Pfasum [4]. Matrices which do not form series such as Gonnet [5, 6], Optima [7], and MIQS [8], can also be assigned to this group.

The matrix constructed on the basis of the model of the Dirichlet mixture of the probabilities of the amino acid background [9] can also be considered evolutionary and, therefore, suitable for aligning sequences whose evolutionary distance between them is not known in advance.

Another branch in the group of evolutionary matrices consists of matrices aimed at comparing sequences of proteins belonging to a particular family [10, 11].

This study was not limited to the evolutionary matrices specially developed for the alignment procedure; hence tests were also conducted on matrices created on the basis of alternative principles, using the alignment quality criterion (see *Methods, Substitution matric*es).

As is known, along with the evolutionary matrices constructed by comparing sequences, among the known amino acid similarity matrices, there are matrices of a different origin. For example, matrices obtained by comparing three-dimensional structures [12–14], as well as those obtained on the basis of the physical and chemical properties of amino acid residues.

In addition, there are known contact energy matrices that reflect the statistics of pair interactions in a protein globule [15].

### Evolutionary distances of matrices and amino acid sequences

When choosing a matrix for the alignment procedure, it is necessary to take into account the relationship between the evolutionary distance separating the sequences and the evolutionary distance to which this matrix corresponds. The question is: should the exact correspondence between the evolutionary distance of the matrix and amino acid sequences be fulfilled or should a deviation in one direction or another be permissible? Therefore, it is necessary to consider all possible combinations of evolutionary distances characterizing matrices and sequences.

As for the origin of the matrixes of the weights of substitutions, according to the hypothesis of Benner et al. [16], for sequences separated by a small evolutionary distance, amino acid substitutions are determined by the genetic code. Due to the structure of the code, substitutions of physico-chemically dissimilar amino acids, i.e. “bad” substitutions (for example, R-W, R-C), but the situation as a whole will not be fatal, since there are few such substitutions, due to the small number of events (substitutions), obviously, as a result of selection.

For sequences separated by a large evolutionary distance, the total number of events is large, but the number of “bad” mutations should remain quite small, as a result of which the overwhelming number of substitutions must occur while maintaining the similarity of physical and chemical properties. Therefore, despite the large number of substitutions (low degree of homology), these substitutions are not fatal.

Thus, it is of interest to know how universal the matrices are, what combinations of evolutionary distances of compared sequences and matrices are optimal, as well as comparisons of the efficiency of matrices obtained on the basis of sequences or three-dimensional structures.

### Matrix efficiency in terms of alignment quality and matrix correlation coefficient

As a possible additional way of predicting matrix efficiency by evaluating a formal measure of the similarity of two matrices, we used the correlation coefficient. First of all, it was necessary to examine whether there is a relationship between the high correlation coefficient of the substitution matrices and the coincidence of alignment quality obtained using these matrices.

### Evolution modeling as a way to evaluate matrix performance

To evaluate the efficiency of the matrices, it is considered advisable to carry out the alignment of sequences of various origins. We used both model sequences constructed according to the scheme of divergent evolution [17] (see *Methods, Test sequences*), and real sequences from Balibase [18, 19]. To generate model sequences, we chose the Dayhoff evolution model [1] for various evolutionary distances.

Thus, in our work, we propose an evolutionary model where a random occurrence of mutations in an arbitrary position of the sequence without any restrictions is assumed. This means that, along with the sequence, the structure of the protein can also change. An example of an evolutionary model involving the preservation of fold is presented in [20]. The main features of the given model are the loss of recognizable similarity of the mutated sequence obtained as a result of long evolution, with the original sequence, while maintaining, basically, the original structure. The model includes restrictions on the introduced mutations in order to preserve thermodynamic characteristics of the protein, such as hydropathy, on which the stability of the overall structure depends.

## Results

In general, the alignment procedure is determined by the substitution matrix and the penalty function, which determines the contribution of insertion-deletions to the weight of an optimal alignment. In our study, we used a linear penalty function, which includes two parameters – a penalty for opening and continuing of insertion-deletion. We performed alignment with local [21] and global [22] algorithms. The quality of algorithmic alignment was evaluated by two parameters (see *Methods, Alignment quality assessment*).

**Fig. 1:**
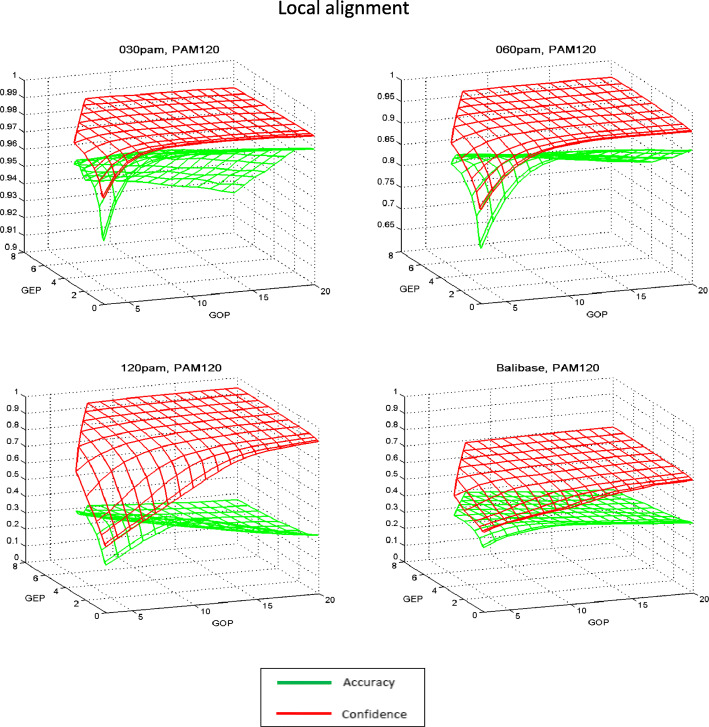
Dependence of the local alignment quality indicators *Accuracy* and *Confidence* with the PAM120 matrix on the values of the penalty function parameters. In the 120 PAM test set, the difference between the values of the two quality indicators reaches the largest value

**Fig. 2:**
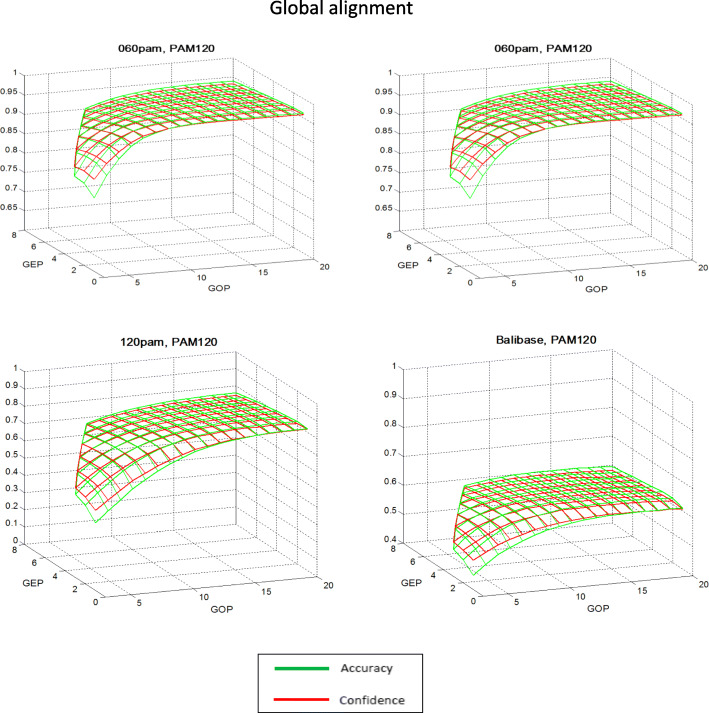
Dependence of the global alignment quality indicators with the PAM120 matrix on the values of the penalty function parameters. In all test sets, the absolute value of the difference between the *Accuracy* and *Confidence* values ​​is noticeably smaller than in the case of local alignment, and varies little over most of the region of the penalty function parameters

**Fig. 3:**
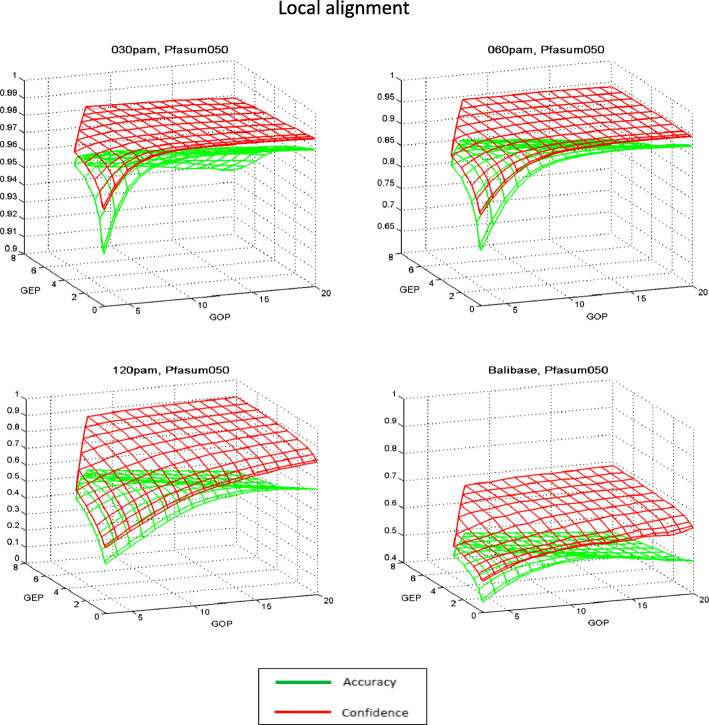
Dependence of the quality indicators of local alignment on the values of the penalty function parameters with the matrix Pfasum050. As in the case of the PAM120 matrix, at all points of the penalty function parameter area, *Accuracy* values exceed *Confidence* values. But the difference between *Accuracy* and *Confidence* in the case of Pfasum050 is less than in the case of PAM120 matrix

**Fig. 4:**
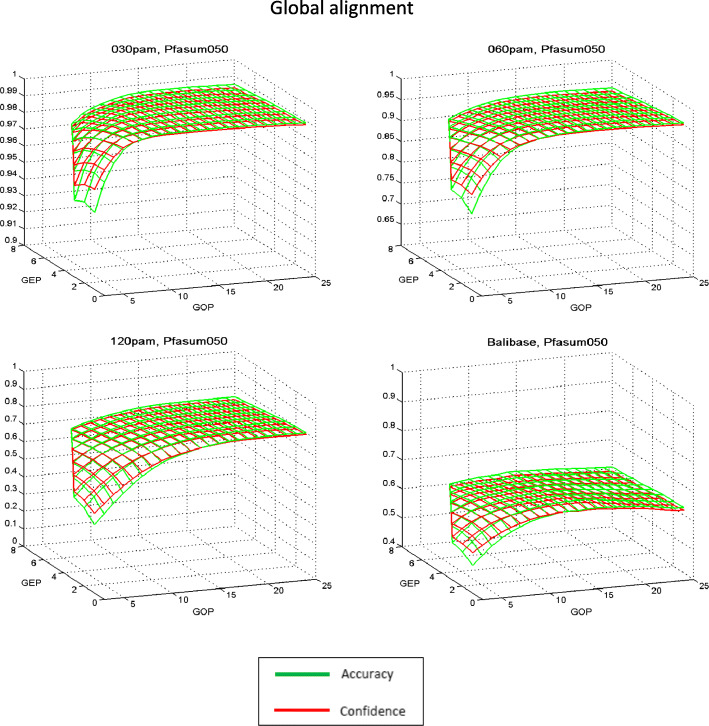
Dependence of global alignment quality indicators on the values of the penalty function parameters with the Pfasum050 matrix

General tendencies of the quality changes in the alignment studied for the majority of the matrices, depending on the type of algorithm and the values of the penalty function, are presented in the example of PAM120 and Pfasum050 matrices in Figs. 1, 2, 3 and 4. In the case of local alignment in all test sets, the numerical values of *Accuracy* are higher than *Confidence* at all points of the penalty function parameter area. Moreover, the difference between the characteristics of alignment quality for small values of the penalty function parameters is rather small, and for the highest values of the penalty parameters (GOP = 20, GEP = 8) this difference attains a significant value. This means that the number of comparisons in the reference alignment is greater than in the algorithmic local alignment for all values of penalty parameters. At the same time, for the global alignment the difference between *Accuracy* and *Confidence* values is significantly smaller, which indicates a negligible difference in the number of comparisons in the reference and in the algorithmic global alignments. Further, in all test sets for small values of the penalty function parameters, the *Confidence* values are greater than the *Accuracy* values, and for the largest values of the penalty parameters, this difference changes its sign.

The selection of an optimal value of penalty parameters was carried out as follows: of the two alignment characteristics (*Accuracy, Confidence*), the minimum was selected, then the maximum was selected from the set of obtained values. The values of the optimal alignment characteristics and the corresponding values of the penalty parameters for all the considered substitution matrices are shown in Tables 1 and 2. A full set of alignment quality values for the entire set of tested parameters is given in Additional files 1, 2: Table S1, Table S2.

**Table 1:** Accuracy and Confidence values of local alignment

Data set		30 PAM				60 PAM		
Matrix	Gap open	Gap extention	Accuracy	Confi-dence	Gap open	Gap extention	Accuracy	Confi-dence
PAM30	19.0	0.2	0.9672	0.9883	8.0	1.0	0.6768	0.8781
PAM60	16.0	0.2	0.9776	0.9881	14.0	0.2	0.8635	0.9507
PAM120	10.0	0.1	0.9816	0.9876	10.0	0.5	0.9244	0.9519
PAM250	8.0	0.1	0.9821	0.9862	11.0	0.2	0.9353	0.9489
Blosum45	10.0	0.1	0.9803	0.9861	14.0	0.2	0.9246	0.9439
Blosum50	14.0	0.1	0.9802	0.9862	13.0	0.5	0.9227	0.9436
Blosum62	13.0	0.1	0.9802	0.9862	17.0	0.5	0.9216	0.9479
Gonnet250	8.0	0.1	0.9828	0.9862	10.0	0.2	0.9329	0.9437
Gonnet_p	7.0	0.1	0.9852	0.9839	8.0	0.1	0.9390	0.9336
Optima	9.0	0.1	0.9814	0.9861	17.0	0.5	0.9138	0.9421
VTML250	9.0	0.1	0.9808	0.9854	11.0	0.1	0.9293	0.9426
MIQS	10.0	0.1	0.9813	0.9860	12.0	0.2	0.9287	0.9433
Pfasum050	10.0	0.1	0.9808	0.9860	14.0	0.2	0.9271	0.9444
Pfasum100	10.0	0.1	0.9800	0.9864	12.0	0.2	0.9200	0.9452
Crooks	11.0	0.1	0.9777	0.9859	14.0	0.2	0.9098	0.9457
CCF53	10.0	0.2	0.9796	0.9862	12.0	0.2	0.9195	0.9453
Moll60	11.0	0.1	0.9793	0.9862	11.0	0.5	0.9142	0.9440
Johnson	15.0	0.1	0.9795	0.9866	17.0	0.5	0.9139	0.9465
Prlic	16.0	0.1	0.9811	0.9853	21.0	0.1	0.9251	0.9394
Blake	23.0	0.1	0.9797	0.9841	25.0	1.0	0.9188	0.9318
Benner	8.0	0.2	0.9803	0.9834	10.0	0.1	0.9197	0.9316
Miyazawa	15.0	0.1	0.9749	0.9821	17.0	0.2	0.8941	0.9307
Data set		120 PAM				Bali Base		
Matrix	Gap open	Gap extention	Accuracy	Confi-dence	Gap open	Gap extention	Accuracy	Confi-dence
PAM30	3.0	1.0	0.1648	0.2826	4.0	1.0	0.3207	0.4471
PAM60	4.0	1.0	0.2781	0.4016	3.0	2.0	0.3928	0.5026
PAM120	6.0	1.0	0.5310	0.6969	7.0	0.5	0.4823	0.5624
PAM250	12.0	0.5	0.7251	0.8039	8.0	0.5	0.5427	0.5821
Blosum45	13.0	0.5	0.6514	0.7664	10.0	0.5	0.5621	0.6121
Blosum50	11.0	1.0	0.6244	0.7482	11.0	0.5	0.5494	0.6050
Blosum62	12.0	1.0	0.5794	0.7378	11.0	0.5	0.5375	0.6019
Gonnet250	11.0	0.5	0.7210	0.7769	8.0	1.0	0.5779	0.6103
Gonnet_p	11.0	0.1	0.7659	0.7538	7.0	0.1	0.6433	0.6338
Optima	10.0	0.5	0.6424	0.7352	9.0	0.5	0.5625	0.6107
VTML250	11.0	0.5	0.7212	0.7757	6.0	1.0	0.5719	0.6025
MIQS	12.0	0.5	0.6969	0.7660	9.0	0.5	0.5729	0.6079
Pfasum050	13.0	0.5	0.6731	0.7602	10.0	0.5	0.5668	0.6090
Pfasum100	9.0	0.5	0.5849	0.7343	7.0	0.5	0.5384	0.5958
Crooks	7.0	1.0	0.4902	0.6733	8.0	0.5	0.5206	0.5958
CCF53	9.0	0.5	0.5819	0.7395	8.0	0.2	0.5358	0.5980
Moll60	7.0	1.0	0.5314	0.6721	8.0	0.5	0.5289	0.6003
Johnson	8.0	2.0	0.4898	0.6623	12.0	0.5	0.5122	0.5917
Prlic	19.0	1.0	0.6714	0.7377	16.0	1.0	0.5741	0.6098
Blake	25.0	4.0	0.6781	0.7396	21.0	1.0	0.5762	0.6016
Benner	12.0	0.5	0.6296	0.6877	8.0	0.5	0.5215	0.5480
Miyazawa	11.0	1.0	0.4928	0.6324	10.0	1.0	0.4826	0.5395

**Table 2:** *Accuracy* and *Confidence* values of global alignment

		30 PAM				60 PAM		
Matrix	Gap open	Gap extention	Accuracy	Confi-dence	Gap open	Gap extention	Accuracy	Confi-dence
PAM30	18.0	2.0	0.9910	0.9907	25.0	2.0	0.9604	0.9600
PAM60	17.0	1.0	0.9916	0.9911	21.0	1.0	0.9610	0.9607
PAM120	13.0	1.0	0.9914	0.9908	13.0	1.0	0.9621	0.9599
PAM250	9.0	1.0	0.9901	0.9894	13.0	1.0	0.9610	0.9575
Blosum45	15.0	1.0	0.9901	0.9893	14.0	1.0	0.9559	0.9531
Blosum50	14.0	1.0	0.9902	0.9895	17.0	1.0	0.9563	0.9533
Blosum62	16.0	1.0	0.9906	0.9900	19.0	1.0	0.9575	0.9549
Gonnet250	11.0	1.0	0.9899	0.9890	11.0	1.0	0.9551	0.9514
Gonnet_p	7.0	1.0	0.9879	0.9864	10.0	1.0	0.9460	0.9398
Optima	10.0	1.0	0.9899	0.9891	14.0	1.0	0.9557	0.9520
VTML250	10.0	1.0	0.9894	0.9885	12.0	1.0	0.9555	0.9515
MIQS	11.0	1.0	0.9897	0.9888	15.0	1.0	0.9564	0.9527
Pfasum050	14.0	1.0	0.9899	0.9890	16.0	1.0	0.9574	0.9542
Pfasum100	11.0	1.0	0.9901	0.9892	14.0	1.0	0.9573	0.9542
Crooks	13.0	1.0	0.9900	0.9893	17.0	1.0	0.9555	0.9524
CCF53	11.0	1.0	0.9900	0.9892	13.0	1.0	0.9563	0.9534
Moll60	12.0	1.0	0.9900	0.9893	16.0	1.0	0.9564	0.9531
Johnson	18.0	1.0	0.9904	0.9899	23.0	1.0	0.9564	0.9547
Prlic	18.0	1.0	0.9896	0.9889	24.0	1.0	0.9532	0.9503
Blake	22.0	1.0	0.9880	0.9879	30.0	1.0	0.9468	0.9458
Benner	9.0	1.0	0.9878	0.9867	12.0	1.0	0.9452	0.9407
Miyazawa	21.0	1.0	0.9864	0.9855	22.0	1.0	0.9432	0.9408
Data set		120 PAM				Bali Base		
Matrix	Gap open	Gap extention	Accuracy	Confi-dence	Gap open	Gap extention	Accuracy	Confi-dence
PAM30	30.0	4.0	0.8065	0.7991	24.0	4.0	0.6257	0.6215
PAM60	26.0	3.0	0.8174	0.8080	20.0	3.0	0.6301	0.6236
PAM120	19.0	1.0	0.8189	0.8152	16.0	2.0	0.6287	0.6205
PAM250	17.0	1.0	0.8325	0.8241	11.0	2.0	0.6310	0.6240
Blosum45	22.0	1.0	0.7949	0.7855	13.0	1.0	0.6495	0.6473
Blosum50	21.0	1.0	0.7925	0.7866	16.0	2.0	0.6523	0.6433
Blosum62	24.0	2.0	0.7972	0.7877	19.0	2.0	0.6533	0.6455
Gonnet250	16.0	1.0	0.8129	0.8037	11.0	1.0	0.6592	0.6541
Gonnet_p	13.0	1.0	0.7870	0.7729	7.0	1.0	0.6470	0.6353
Optima	17.0	1.0	0.7907	0.7830	13.0	1.0	0.6515	0.6463
VTML250	15.0	1.0	0.8110	0.8017	11.0	1.0	0.6544	0.6486
MIQS	18.0	1.0	0.8046	0.7964	13.0	1.0	0.6518	0.6479
Pfasum050	21.0	1.0	0.8015	0.7939	15.0	2.0	0.6599	0.6507
Pfasum100	18.0	1.0	0.7973	0.7891	12.0	1.0	0.6527	0.6499
Crooks	18.0	2.0	0.7921	0.7823	14.0	2.0	0.6553	0.6470
CCF53	18.0	1.0	0.7923	0.7841	14.0	1.0	0.6488	0.6429
Moll60	19.0	1.0	0.7819	0.7762	12.0	2.0	0.6484	0.6403
Johnson	29.0	2.0	0.8011	0.7935	19.0	3.0	0.6524	0.6456
Prlic	13.0	1.0	0.7870	0.7729	18.0	2.0	0.6476	0.6427
Blake	30.0	4.0	0.7608	0.7547	24.0	3.0	0.6444	0.6419
Benner	16.0	1.0	0.7385	0.7282	10.0	1.0	0.5917	0.5848
Miyazawa	30.0	2.0	0.7539	0.7449	20.0	3.0	0.6024	0.5936

The optimal values of the alignment quality (*Accuracy*, *Confidence*) with the corresponding values of the penalty function parameters (GOP, GEP) are given. Data were obtained for all matrices examined, on test sets of the generated sequences: 30 PAM, 60 PAM, 120 PAM, and on Balibase [18] sequences. A full set of alignment quality values for the entire set of tested GOP and GEP parameters is given in Additional files 1, 2: Table S1, Table S2

### Trends in the optimal penalty for opening insertion-deletions depending on the evolutionary distance between sequences

#### Local alignment

Let us consider the tendency of change of optimal penalty for gap opening depending on the increase in the evolutionary distance between the sequences and the increase in the evolutionary distance for which the matrix was constructed, using the example of PAM matrices. Table 1 shows that when using short distance matrices (PAM30, PAM60), with an increase in the evolutionary distance between the compared sequences from 30 to 120 PAM, the optimal gap opening penalty (GOP) decreases. Thus, the optimal GOP values for the PAM30 and PAM60 matrices are (19, 8, 3) and (16, 14, 4) for evolutionary distances of 30, 60, and 120 PAM, respectively.

Further, when aligned with the PAM120 matrix, the decrease in optimal GOP values is not so noticeable (10, 10, 6). Finally, when aligned with the PAM250 matrix, on the contrary, with an increase in the evolutionary distance between sequences, the optimal gap opening penalty increases: GOP = 8, 11, 12.

Other matrices constructed for large evolutionary distances behave similarly to the PAM250 matrix: Gonnet250: GOP = 8, 10, 11; Gonnet_p: GOP = 7, 8, 11; VTML250: GOP = 9, 11, 11; MIQS: GOP = 10, 12, 12.

The remaining matrices give the worst values of accuracy and confidence; hence we did not discuss them.

#### Global alignment

In this case, a simpler pattern was observed: with an increase in the evolutionary distance between sequences, the optimal penalty for gap opening increased (or does not decrease) for almost all tested matrices (see Table 2). As for gap extension penalties (GEP), their value was considered small for both local and global algorithms, and there was a monotonous increase in the penalty with increasing evolutionary distance between sequences.

### Dependence of the alignment quality on the ratio of evolutionary distances between sequences and evolutionary distances for which matrices are designed

#### Local alignment

Consider the case when the evolutionary distances between sequences and the evolutionary distances for which the matrix was constructed do not coincide. As can be seen from Table 1, the alignment quality of sequences separated by a large evolutionary distance using matrices for a small evolutionary distance is significantly lower compared to the case of alignment of the same sequences using matrices for a large evolutionary distance. This property is clearly seen in the example of matrices of the PAM series (see Table 1, test set 120 PAM). Here, the *Accuracy*, *Confidence* alignment quality indicators for the PAM250 matrix are approximately 4 and 3 times higher compared to the PAM30 matrix. A similar dependence was observed for matrices of the Blosum series. It should be noted that the considered Blosum matrices cover a narrower range of evolutionary distances, and therefore, their efficiency changes less depending on the matrix number. When aligning sequences from the 120 PAM test set, the Blosum45 matrix is most effective, followed by Blosum50 and Blosum62. For Balibase sequences, this trend persists, but is less noticeable. Note that, unlike PAM matrices, a lower Blosum matrix number corresponds to a larger evolutionary distance.

On the contrary, the quality of alignment of sequences spaced a short evolutionary distance using matrices for a large evolutionary distance is not lower (and in some cases slightly higher) than when matrices are used for a small evolutionary distance. For example, on the 30 PAM test set, the alignment quality using the PAM250, Gonnet250, and Gonnet_p matrices is slightly higher compared to the PAM60 matrix (see Table 1).

#### Global alignment

For this type of alignment, in the case of remote sequences, the advantage of matrices for long distance over matrices for short distance is not as significant as in the case of local alignment. Therefore, on the 120 PAM test set (see Table 2), the PAM250 matrix shows the best result, followed by the PAM120 and PAM60 matrices. The PAM30 matrix is only slightly inferior to the Gonnet250 and VTML250 matrices. This superiority of the PAM matrices for global alignment on this test set is most likely due to the evolutionary model used (see *Methods, Test sequences*). The efficiency of the considered matrices of the Blosum series on the 120 PAM test set, as well as on Balibase sequences, is practically independent of the matrix number.

Meanwhile, the quality of global alignment of sequences with high homology is even less dependent on the type of substitution matrix than in the case of local alignment. On the 30 PAM test set (see Table 2), the PAM60 matrix shows the best result, followed by the PAM120, PAM30, PAM250 and the Blosum series matrices. However, there is an insignificant difference in the quality of indicators.

Thus, the universality of the matrix for a large evolutionary distance in terms of the alignment efficiency of sequences with different evolutionary distances is revealed both with local and global alignment. However, with local alignment, the advantage of the matrix for large distances is greater [6].

### Efficiency of matrices of various evolutionary distances in the case of local or global alignment

Consider the general quantitative patterns found in the previous section.

PAM matrices exhibited the greatest dependence on algorithm type. Therefore, when aligning the sequences of the test set 120 PAM using the PAM30 matrix, the ratio of the quality parameters of global and local alignments *Accuracy* and *Confidence* were approximately 5 and 3, respectively; for the PAM60 matrix, these ratios were approximately 3 and 2. For the PAM120 matrix, these ratios were about 1.5 and 1.2, and for the PAM250 matrix, they were close to one (see Table 3, test set 120 PAM).

**Table 3:** The ratio of local and global alignment quality parameters on 120 PAM test suite sequences and Balibase sequences

Matrix	120 PAM		Balibase	
	Global / Local		Global / Local	
	Accuracy	Confidence	Accuracy	Confidence
PAM30	4.8938	2.8277	1.9510	1.3901
PAM60	2.9392	2.0120	1.6041	1.2407
PAM120	1.5422	1.1698	1.3035	1.1033
PAM250	1.1481	1.0251	1.1627	1.0720
Blosum45	1.2203	1.0249	1.1555	1.0575
Blosum50	1.2692	1.0513	1.1873	1.0633
Blosum62	1.3759	1.0676	1.2154	1.0724
Gonnet250	1.1275	1.0345	1.1407	1.0718
Gonnet_p	1.0275	1.0253	1.0058	1.0024
Optima	1.5409	1.3453	1.6990	1.5589
VTML250	1.1245	1.0335	1.1443	1.0765
MIQS	1.1545	1.0397	1.1377	1.0658
Pfasum050	1.1908	1.0443	1.1643	1.0685
Pfasum100	1.3631	1.0746	1.2123	1.0908

Thus, with increasing evolutionary distance for which PAM matrices are designed, their performance for local alignment approaches that of global alignment. The same trend was observed for alignments of Balibase sequences.

The considered Blosum matrices showed a similar tendency, but it was less pronounced, since the evolutionary distance between Blosum62 and Blosum45 was less than the evolutionary distance between PAM30 and PAM250 (see Table 3).

On the 120 PAM test set, the matrices Gonnet_p, Gonnet250 and VTML250 showed the least dependence on the type of alignment algorithm; on Balibase sequences, the least dependence on the type of algorithm was shown by the Gonnet_p, MIQS, and Gonnet250 matrices.

Thus, matrices designed for a greater evolutionary distance and for providing better alignment quality also show less dependence on the type of algorithm.

## Discussion

### Interpretation of matrix test results

Let us explain the observed dependencies, taking into account the specifics of the substitution matrices for different evolutionary distances and the differences in sequences separated by one or another evolutionary distance.

Matrices for small evolutionary distances are characterized by a relatively large value of diagonal elements (from 6 to 13) and a small value outside the diagonal elements (from − 15 to 2 for the matrix PAM30). This is because matrices for small evolutionary distances are designed to compare sequences containing a large proportion of matching characters. The alignment of sequences spaced a small evolutionary distance should have many symbol mappings and few deletion inserts, and to eliminate the occurrence of unnecessary insertion-deletions, a large penalty value is necessary (GOP about 19.0). At the same time, when aligned using both global and local algorithms, due to the large number of matches, maximum goal function is achieved over the entire length of the sequences (without dropping areas of low homology), and local alignment practically coincides with the global one. Since global alignment is the reference in our model for all evolutionary distances and types of algorithms, it gives a good quality of alignment.

In the case of alignment of distant sequences with a local algorithm with the matrix for small evolutionary distances, a large value of the penalty which leads to the maximum goal function will be found, most likely on the fragment than on the whole sequence, which results in a mismatch with the reference global alignment.

The fact that, when aligning with a local algorithm with large values of the penalty function, alignments of the local type are obtained, leads to a much smaller number of comparisons in the algorithmic alignment compared to the standard alignment. This is equivalent to the inequality *Accuracy* < *Confidence* (see *Methods, Alignment quality assessment*), which is clearly illustrated in Figs. 1 and 3.

When considering the population of the aligned pairs, we can see that the low value of the average accuracy and reliability is the result of a large number of pairs with a complete mismatch with the reference alignment. When aligned using local algorithm, by reducing the penalty, as the calculation shows, it is possible to obtain the alignment of the global type, but with a large number of gaps and significantly different from the reference (*Accuracy* = 0.165, *Confidence* = 0.283, see Table 1, test set120PAM, matrix PAM30). The observed inequality *Accuracy* < *Confidence* indicates a trend in the difference between algorithmic and reference alignments, expressed in a significant number of local type alignments in a set of algorithmic alignments.

Matrices for a large evolutionary distance are characterized by a relatively smaller value of the diagonal and a larger value outside the diagonal elements (2..12 and − 8..7 for PAM250). The local alignment of sequences spaced by a small and medium evolutionary distance, with such matrices and gap opening penalty< 12, has enough similarities with global alignment, which explains the high similarity with reference alignment (average *Accuracy* > 0.93, *Confidence* > 0.94). With a large evolutionary distance between sequences (120 PAM), with penalties of GOP > 3, GEP > 2, the alignments obtained by the local algorithm have less similarity to the global alignment. However, the trend of increasing optimum value GOP with increasing evolutionary distances between sequences was observed.

Global alignment is characterized by an increase in the optimal penalty for gap opening with increasing distance between sequences. This trend is present in trials with all matrices. This is due to the fact that in the applied evolution model with increasing distance, the increase in the number of insertions-deletions is slower than the accumulation of mutations.

Global alignment is characterized by an increase in the optimal penalty for opening an insertion-deletion with an increase in the distance between sequences. This trend is present in trials with all matrices. This is explained by the fact that in the applied model of evolution with increasing distance, the rate of increase in the number of insertions is slower than the accumulation of mutations.

Thus, we concluded that matrices corresponding to large evolutionary distances are universal. They not only align sequences best separated by large evolutionary distances, but align evolutionarily close sequences no worse than they align with matrices of the corresponding evolutionary distance. Calculations showed that among matrices corresponding to a large evolutionary distance, the Gonnet250 matrix gives the most stable alignment quality. The Gonnet_p matrix [6] obtained from this matrix gives good alignment quality in the case of a local algorithm. The high PAM250 matrix score on the 120 PAM test set can be largely due to the evolutionary model used (see *Methods*). The ranking of the considered matrices by the average value of two parameters of the alignment quality is given in Table 4.

**Table 4:** Matrices ranging by alignment quality

Local Alignment				Global Alignment			
120 PAM		Balibase		120 PAM		Balibase	
PAM250	0.7645	Gonnet_p	0.6386	PAM250	0.8283	Gonnet250	0.6567
Gonnet_p	0.7599	Gonnet250	0.5941	Gonnet250	0.8083	Pfasum050	0.6553
Gonnet250	0.7490	MIQS	0.5904	VTML250	0.8064	VTML250	0.6515
VTML250	0.7485	Pfasum050	0.5879	MIQS	0.8005	MIQS	0.6499
MIQS	0.7315	VTML250	0.5872	Pfasum050	0.7977	Optima	0.6489
Pfasum050	0.7167	Optima	0.5866	Optima	0.7869	Gonnet_p	0.6412
Optima	0.6888	PAM250	0.5624	Gonnet_p	0.7800	PAM250	0.6275

Matrices are presented that show the best quality of local and global alignment on the **120 PAM** and **Balibase** test sets. Ranking in descending order is based on the average value of the *Accuracy* and *Confidence* parameters

### Investigation of the relationship between the correlation coefficient and matrix performance

The use of simple methods for comparing matrices, such as calculating the correlation coefficients a priori, seems unpromising. Indeed, a comparison of the Table of correlation coefficients (see Additional file 3: Table S3) with the data on alignment quality (Tables 1 and 2) does not allow us to reveal obvious trends in the relationship between the alignment quality and the correlation coefficients.

Naturally, matrices belonging to the same family (e.g., PAM, Blosum) show high correlation coefficients.

Further, it is significant that large distance matrices of evolutionary type, such as PAM250, Gonnet, MIQS, VTML250, and Pfasum050, recorded the highest correlation coefficient between each other (0.895 < *r* < 0.995), over the entire sample of matrices considered, despite the fact that the methods of their construction and initial data are different. It should be recalled that these same matrices showed the best alignment quality.

The situation is different with matrices obtained on the basis of structural alignment (Johnson, Prlic, Blake). Here, on the contrary, in the case of Johnson and Prlic matrices, the maximum correlation coefficient for the entire sample was achieved with matrices of evolutionary origin (Blosum50, Ppfasum050, respectively). The Blake matrix recorded the best correlation with the Johnson matrix. Thus, the correlation was partial, the generality of trends was not observed.

More complex dependencies were also observed. Since the structure was more conservative than the sequence, it can be assumed that matrices based on structural alignment are suitable for aligning sequences separated by large evolutionary distances. From Table 2 (test set PAM120), it follows that this assumption is valid for the Johnson matrix in the case of the global algorithm (*Accuracy* = 0.8011, *Confidence* = 0.7935), but it does not hold for the local algorithm for which the quality of alignment is noticeably lower (*Accuracy* = 0.4898, *Confidence* = 0.6623, see Table 1, test set PAM120). In contrast, for the Prlic and Blake matrices, the alignment quality characteristics for the global and local algorithms are quite close: (0.6714, 0.7377) for the Prlic matrix and (0.6781, 0.7396) for the Blake matrix in the case of the local algorithm, and accordingly (0.7870, 0.7729), (0.7608, 0.7547) - for the global algorithm (Tables 1, and 2, test set PAM120).

It should be noted that the correlation coefficients of the above-mentioned structural matrices with large distance evolutionary matrices (Pfasum050, Optima) are also quite high. Nevertheless, it would be incorrect to identify the tendency of coincidence in the quality of alignment with a high correlation coefficient. The correlation coefficient score cannot compete with the alignment quality criterion.

## Conclusion

In this paper, we investigated the correspondence between the evolutionary distances of amino acid substitution matrices and the sequences to be aligned. It has been shown that, although at first glance one can limit the use of matrices with an evolutionary distance coinciding with the assumed evolutionary distance between sequences, a complete study of all combinations of matrices and sequences results in nontrivial conclusions. It was shown that the result of the alignment of sequences is separated by a greater evolutionary distance than the evolutionary distance of the substitution matrix. This shows a significant discrepancy between algorithmic and reference alignment.

Conversely, in the case when the evolutionary distance between sequences is less than the evolutionary distance of the matrix, the coincidence of the obtained alignment with the reference is much higher.

So for the main criterion of the quality of alignment, matrices corresponding to large evolutionary distances have a significant advantage. In contrast to the matrices for close distance, matching mainly equal coincident symbols in the alignment, matrices for long distance are able to match a. a. residues assembled by physical and chemical properties. Thus, not the literal coincidence of characters is preserved, but physical and chemical properties, of which hydrophobicity is the main one, as shown in [20].

We examined the following question: Is there a relationship between the high correlation coefficient of the substitution matrices and the coincidence of alignment quality obtained with these matrices? It was noted that matrices corresponding to a high evolutionary distance, for which a high level of alignment quality is achieved, show a higher correlation coefficient between each other. Further, matrices corresponding to a small evolutionary distance, showing poor alignment quality, are characterized by lower correlation both between themselves and with matrices corresponding to a large evolutionary distance. Nevertheless, it would be incorrect to always identify the tendency of coincidence in alignment quality with a high correlation coefficient. Estimation by the correlation coefficient cannot compete with the criterion by the quality of alignment.

The results obtained can serve as a recommendation for the practical use of the alignment procedure, especially in those cases when the evolutionary distance between sequences cannot be estimated.

## Methods

### Test sequences

To evaluate the performance of substitution matrices for different evolutionary distances, the alignments of sequences of various origins were performed. We used both model sequences corresponding to different evolutionary distances and a sample of real pairs of sequences from Bali base [18, 19].

The test sets of generated model sequences contained 1000 pairs of amino acid sequences in a 20-letter alphabet. Three sets were constructed using the same methodology, which differ in the values of evolutionary distance. Three sets were constructed using the same methodology, which differ in the values of the evolutionary distance. In the test pair, both sequences *S*1 and *S*2 were generated from the original sequence *S*0 (“ancestor”). Thus, the process of generating a test pair consisted of two steps: generating a common ancestral sequence; generation of test sequences in accordance with the value of the *PAM* parameter (*PAM* = 30, 60, 120) [1].

The ancestral sequence *S*0 was generated as a random Bernoulli sequence with a length of 200 a.a., comparable with the typical length of the polypeptide chain, with the frequency of amino acids occurrence in accordance with [1].

According to the same procedure, the sequences *S*1 and *S*2 were constructed independently of one another. This procedure consisted of two stages. At the first stage, insertions and deletions were introduced into the ancestral sequence. For this, each position of the ancestral sequence *S* was checked for the occurrence of an insert before this position or deletion, starting from this position, with the probability of an event, according to [23]:

$$ P\ (indel)=0.0224-0.0219\cdotp {e}^{\left(-0.01168\bullet PAM\right)}, $$

where *PAM* is a number characterizing the evolutionary distance between the ancestral and mutant sequences.

The insertion or deletion length was randomly selected from the Zipf distribution, which, according to [23], does not depend on the value of the evolutionary distance.

**Fig. 5:**
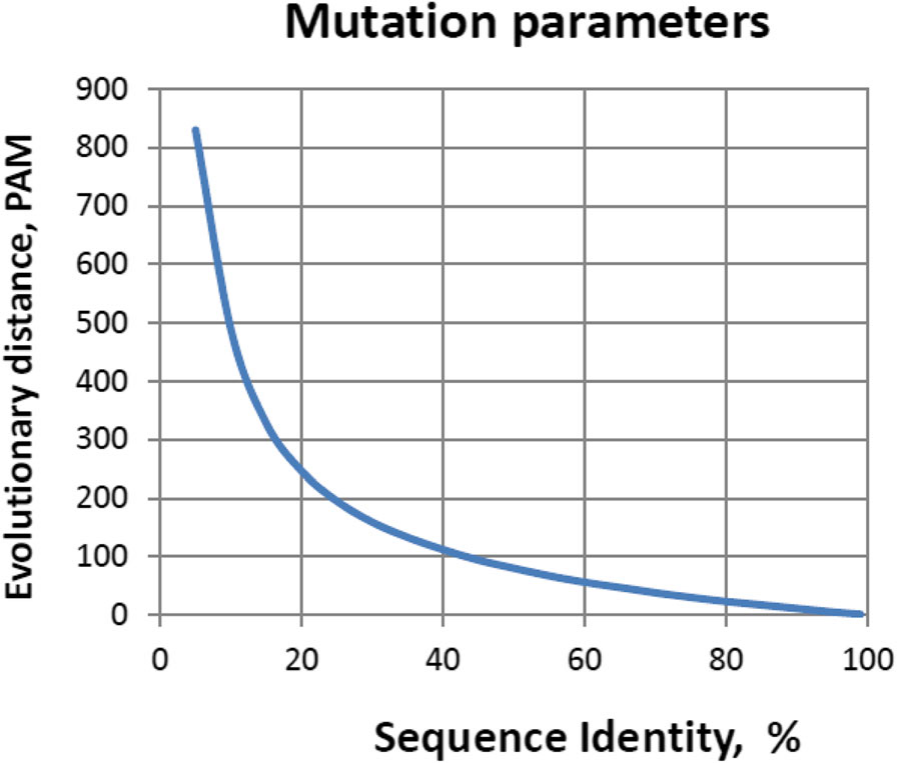
Correspondence of the values of the PAM parameter to the average percentage of identity in the positions of the ancestral and mutant sequences (%*id*). In a mutant sequence, only substitutions are allowed. Note that the maximum *PAM* value shown in Table 23 of [1] is 328 and corresponds to %*id* = 15%. For %*id* values less than 15%, *PAM* values are added by polynomial approximation at six points

At the second stage, point mutations were introduced into the sequence obtained at the first stage. Thus, mutations were introduced only in areas left over from the ancestral sequence. One cycle of introducing mutations was that in each position with a certain probability a substitution can be made, while the probability of a new symbol in this position is determined by the probability matrix *PAM*1 [1]. This cycle was repeated a number of times equal to the value of the PAM parameter. Figure 5 shows a typical relationship between the average percentage of matches between the ancestral and mutant sequences (% *id*) and the value of the *PAM* parameter (according to Tables 2 and 3 from [1]). The value of the share of matches (*id*) for two random sequences with an alphabet of length *n* and the same distribution of frequencies of characters occurrence (*f*_1_, …,*f*_*n*_) in the first and second sequences is defined as:

$\sum \limits_{i=1}^n\frac{1}{f_i^2}$ (see for example [24]).

For the distribution of amino acid frequencies from Dayhoff et al. [1] this value is 0.0601 and lies at the left border of the domain of definition of this function. The area of highest growth of the first derivative of the function falls on the so-called “twilight zone”, corresponding to the values of sequence identity 20% ≤ *id* ≤ 35% according to [6], or 10% ≤ *id* ≤ 30% according to [25].

To determine the evolutionary distance between the modified sequences, on the basis of these alignments, the alignment of sequences S1 and S2 was constructed in such a way that positions originating from a common ancestor in sequence S0 were compared to each other.

**Fig. 6:**
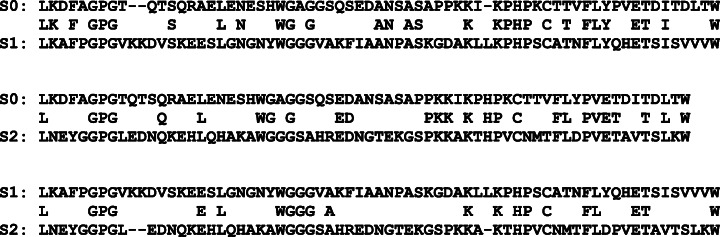
Using fragments of pairwise alignments as an example, we present a scheme for generating a reference alignment in accordance with the model of divergent evolution. Here, the pairs (*S*0, *S*1) and (*S*0, *S*2) denote the generated alignments of the ancestral sequence *S*0 with the mutant sequences *S*1 and *S*2 generated from it, corresponding to an evolutionary distance of 120 PAM; (S1, S2) is the resulting pair alignment corresponding to the scheme of divergent evolution Further, for simplicity, the distance between *S*1 and *S*2 is denoted by the corresponding distance of sequential evolution (in this example, 120PAM), although the true distance between *S*1 and *S*2 is almost two times greater (see Table 5).

An example of text sequences shown in Fig. 6 illustrates the obtaining of alignment corresponding to the scheme of divergent evolution from two alignments of sequential evolution.

Table 5 shows the values of alignment parameters obtained for two evolutionary schemes. As can be seen from Table 5, the reference alignment obtained by superimposing the second rows of pairs has different PAM values defined for mutations and deletion insertions (columns *PAM* (*id*) and *PAM* (*indel*)). This is due to the fact that with a sufficiently large number of accepted substitutions, the proportion of “visible” substitutions decreases.

**Table 5:** *Id* and *Indel* values for sequential and diverging evolution models

Test sets parameters		Sequential evolution					
		id	id	id	indel	indel	indel
N	PAM	(S0, S1)	(S0, S2)	Average	(S0, S1)	(S0, S2)	Average
1000	**30**	0.7481	0.7499	0.7490	0.0324	0.0343	0.0334
1000	**60**	0.5788	0.5796	0.5792	0.0526	0.0560	0.0543
1000	**120**	0.3758	0.3770	0.3764	0.0740	0.0754	0.0747
10,000	**30**	0.7504	0.7501	0.7503	0.0330	0.0336	0.0333
10,000	**60**	0.5804	0.5807	0.5806	0.0526	0.0511	0.0519
10,000	**120**	0.3767	0.3762	0.3765	0.0755	0.0762	0.0759
Test sets parameters		Divergent evolution					
		id		PAM (id)	indel	PAM (indel)	
N	PAM	(S1, S2)		(S1, S2)	(S1, S2)	(S1, S2)	
1000	**30**	0.5793		**60.55**	0.0662	**68.56**	
1000	**60**	0.3756		**122.25**	0.1072	**202.14**	
1000	**120**	0.2009		**245.08**	0.1473	**> 830**	
10,000	**30**	0.5804		**60.31**	0.0661	**68.30**	
10,000	**60**	0.3773		**121.53**	0.1023	**170.52**	
10,000	**120**	0.2025		**243.45**	0.1490	**> 830**	

*N* is the number of sequences in the test set, *id* is the identity fraction*, indel* is the proportion of insertion-deletions. Rows correspond to different values ​​of the PAM parameter. In the **“Sequential evolution”** block, the *id* (*S*0, *S*1) and *id* (*S*0, *S*2) columns show the proportion of matching positions in the alignments of the ancestor sequence with the descendant sequences; the *indel* (S0, S1) and *indel* (S0, S2) columns show the fraction of inserts for the same sequences. The **“Divergent evolution**” block in the columns *id* (S1, S2) and *indel* (S1, S2) shows the alignment characteristics of descendant sequences. The data are for sets of 1000 pairs of sequences and 10,000 pairs, the results differ by tenths of a percent

In Fig. 5, this is seen in the growth of *PAM* with a decrease in *id*. Thus, in the resulting alignment, the number of “visible” substitutions corresponds to the sum of the substitutions made in the initial alignments, but does not equal the sum of the “visible” substitutions in them. At the same time, the total length of insertions and deletions in the resulting alignment, due to their small length relative to the total length of the sequences, is approximately equal to the sum of deletion inserts in the original alignments (which corresponds to a larger *PAM* value).

Note that in the described procedure, test pairs of sequences were built according to the “descendant1 - descendant2” scheme, and not according to the “ancestor-descendant” scheme. The first scheme better models the comparison of real sequences. However, the *PAM* parameter traditionally used to characterize the evolutionary distance is oriented to the second scheme. Thus, the actual *PAM* value for the second circuit is approximately two times greater than the specified value. This evolutionary model was used by the authors in [8] to generate homologous cores of the compared sequences. Earlier, a similar method of modeling evolution was applied in [6, 26].

### Alignment quality assessment

We were interested in how close algorithmic alignments are to reference alignments, i.e. alignments in which positions originating from the same position of the ancestral sequence are matched. To assess the degree of this proximity (“alignment quality”), we used the *Accuracy* and *Confidence* measures described in [6, 25, 27]:

*Accuracy = I/R, Confidence = I/A*, where *I* is the number of matching comparisons in the reference and algorithmic alignments, *R* is the total number of comparisons in the reference alignment, *A* is the total number of comparisons in algorithmic alignment.

### Alignment

The alignment of all sequences was performed by two algorithms: the local Smith – Waterman algorithm [21], and the global Needleman-Wunsch algorithm [22] with an affine penalty function for insertion-deletions. The parameters of the penalty function were tested as follows: the penalty for gap opening (GOP) ranged from 3 to 30, while the penalty for gap extension (GEP) ranged from 0.1 to 8.

### Substitution matrices

We examined matrices of various types and origin in order to evaluate their performance in restoring the reference alignment, i.e. one in which positions originating from the same position in a common ancestral sequence are matched. Table 6 lists the tested matrices.

**Table 6:** Tested substitution matrices

Matrix	Reference	Description
*Evolutionary matrices*		
PAM 30, 60, 120, 250	Dayhoff et al. [1]	Evolutionary model of point substitutions
Blosum45, 50, 62	Henikoff et al. [2]	Series based on the alignment of segments of related sequences from protein families grouped into blocks
Gonnet250	Gonnet et al. [5]	Matrix based on substitutions in protein families on an extended database for long evolutionary distances
Gonnet_p	Vogt et al. [6]	Later modification of Gonnet250
Optima	Kann et al. [7]	Detection of differences between homologues and non-homologues for a large evolutionary distance
VTML250	Muller et al. [3]	Improved evolutionary model based on maximal likelihood method (for distant homologue detection)
MIQS	Yamada et al. [8]	Data derived on the basis of principal component analysis of the previously known matrices (Blosum, VTML, Benner)
Pfasum 50, 100	Keul et al. [4]	Model based on modern data covering a large and diverse sequence space.
*Matrix based on Dirichlet mixture model*		
Crooks	Crooks et al. [9]	The model takes into account the difference in the dynamics of substitutions depending on the time of evolution.
*Evolutionary matrices for special protein families*		
CCF53	Brick et al. [10]	Search for homologues in families of related proteins, taking into account the bias of the amino acid composition characteristic for proteins of two species of the genus *Plasmodium.*
MOLLI60	Lemaitre et al. [11]	General method for constructing matrices focused on a certain bias of amino acid composition, based on the example of bacteria proteins of the *Mollicutes* class.
*Matrices based on the structural alignment*		
Johnson	Johnson et al. [12]	Obtained by calculating the substitutions of amino acid residues in the structural alignment of proteins from homologous families with a low level of sequence identity.
Prlic	Prlic et al. [13]	Obtained on the basis of superposition of pairs of proteins having a similar structure, but low sequence identity.
Blake	Blake et al. [14]	Based on structural superposition data, taking into account differences in arrays of amino acid residues substitutions for distant and closely related homologs.
*Genetic code matrix*		
Benner	Benner et al. [16]	Based on the number of nucleotide substitutions required for a given amino acid substitution.
*Contacts energy matrix*		
Miyazawa	Miyazawa et al. [15]	Based on the assessment of the distribution of contacts in three-dimensional protein structure.

Further, the matrices considered are described in more detail.

### Evolutionary matrices

#### Pam [1]

Point accepted mutations matrix series became the first kind of evolutionary matrix. In order to obtain it, Dayhoff et al. estimated amino acid substitutions in 72 groups of closely related proteins (id> 85%), consisting of more than 1300 sequences. PAM1 matrix was obtained as a result of the calculation of amino acid residues substitutions frequencies. It corresponds to an evolutionary distance unit of 1PAM, at which 1% of residues are substituted. The remaining PAM matrices were obtained from the original PAM1 matrix by raising it to the power, which is the value of the evolutionary distance. The remaining PAM matrices can be obtained from the original PAM1 matrix due to its exponentiation, which is also an evolutionary distance value. Thus, matrices with a larger number correspond to a larger evolutionary distance. Note that with an increase in the number of the PAM matrix, the relative value of the diagonal terms decreases and approaches the average value of the off-diagonal terms. Further, from the substitution frequency matrices, the substitution weight matrices were obtained, whose elements are the logarithm of the ratio of the substitution frequency to the product of the frequencies of occurrence of the corresponding amino acids.

#### Blosum [2]

Blosum series matrices were developed on the basis of multiple comparison (alignment without gaps) of relative motifs segments contained into blocks. Single block presents conservative protein series zone as relative segments of individual proteins, which are located one below the other. In other words, this single block presents a two-dimensional array, where each line is a protein sequence segment, and each column shows the position of leveled balance.

In total, about 2000 blocks of aligned sequence segments were defined characterizing more than 500 groups of related proteins. Then these blocks were combined into groups in accordance with the identity of their segments. To decrease the dependence on amino acid matching frequency, these sequences were grouped in the clusters inside blocks. Each cluster was considered as one sequence, when matching of pairs was calculated. Based on the substitutions frequency in each group, its own Blosum matrix was built. A matrix number means the amount (percentage) of identities, which is typical for the group.

Thus, unlike PAM matrices, Blosum series matrices are obtained on the basis of direct data, and not by extrapolation from a small evolutionary distance to a large one. In this case, a larger number corresponds to a smaller evolutionary distance.

#### Gonnet250 [5]

Gonnet250 matrix was developed with almost the same methods as PAM series matrices, but significantly larger data sets were used. This provides it with better validity compared to PAM matrices. Therefore, the total length of the sequences from the database was more than 8 million amino acid residues. Each sequence was compared with the entire database, as a result of which 1.7 million alignments were obtained. On this basis, a matrix was constructed corresponding to the evolutionary distance of 250 PAM.

#### Gonnet_p [6]

The matrix was obtained from the Gonnet250 matrix by increasing all its elements by a constant value to get positive values for all elements of the resulting matrix.

#### Optima [7]

This evolutionary matrix can be interpreted as the matrix of the “third generation”. To create a new matrix intended to remote homologues recognition, a test set based on the Cluster of Orthologs Groups (COG) [28] was built. This was unlike Gonnet and PAM series matrices, which were built on the basis of substitution frequencies obtained for all considered sequences from the database. Based on the alignment of homologous and non-homologous sequences, the weight function was optimized to distinguish between homologous and non-homologous pairs.

#### VTML [3, 29]

The matrices of the VTML series are a later development of the model originally implemented in the matrices of the PAM series [1] and then continued by such matrices as [5, 7]. The VTML matrix series was originally designed to better detect remote homologs, but is also used to build high quality multiple alignments [30]. VTML matrices were constructed by iteratively estimating evolutionary distances and substitution rates from a set of pairwise sequence alignments using maximum likelihood estimation. In order to build the initial matrix approximation, the Dayhoff model was used. Pairwise alignments were obtained by random selection of two pre-aligned sequences from each protein family of the SYSTERS database [31]. This data set is much larger and more diverse compared to the set on the basis of which PAM matrices were obtained, which allows VTML matrices to provide more reliable detection of remote homologs.

#### MIQS [8]

This matrix is based on information associated with existing matrices condensed into a new matrix that can detect more distant homologues. For this purpose, matrices of Blosum, and VTML series; designed by Benner et al. [16] were selected. The new matrix was developed by applying principal component analysis to the existing matrices, using the appropriate benchmarks SCOP [32], and CATCH [33]. Based on these data, a special test set, CATH20-SCOP, consisting of 1754 sequences, was built. The resulting matrix was tested using SSEARCH. Comparison was made with existing general purpose matrices.

#### Pfasum [4]

Matrices of this series were obtained from multiple alignments of the sequences of seeds of the families of the Pfam protein database (version 29.0) [34], which are a small set of representative members of each family. Each multiple sequence alignment in the Pfam seed dataset was processed separately, the calculated substitution frequencies were accumulated in a separate matrix and then subsequently converted to final rounded values.

Thus, Pfasum matrices are based on modern data, covering a large and diverse sequence space of 47.3 billion amino acid pairs in 16,295 multiple alignments. In addition, most existing substitution matrices are derived only from highly conservative or filtered sequence data by excluding regions containing gaps or ambiguous amino acids. By contrast, the PFASUM matrices design takes into account all information.

### Matrix based on Dirichlet mixture model

#### Crooks [9]

This research was inspired by the fact that the dynamics of substitutions in amino acid sequences at large and small evolutionary distances are different. Previously, this matter was considered in [3, 5, 16]. The problem is that the PAM and VTML series matrices were built on the assumption that the initial distribution of amino acids in the sequences is the same in all positions; in reality, it may differ, while remaining stable in a separate position, over a long evolutionary time. A dynamic model of amino acid substitutions is proposed, which suggests that each site in the protein sequence has its own amino acid background, which in turn, fits the distribution of “backgrounds”.

### Evolutionary matrices for special protein families

#### CCF53 [10]

The aim of this study was to improve the search for homologues in families of related proteins, taking into account the bias of the amino acid composition characteristic for proteins of specific species. For this purpose, a substitution model based on “fuzzy” clustering is proposed, in contrast to the hierarchical clustering used in the construction of the Blosum series matrices. To calculate the matrices, 1834 multiple alignments were used from the BLOCKS database, corresponding to the amino acid composition characteristic of the proteins of two species of the genus *Plasmodium*. The use of these matrices reduces the number of false positive hits when searching for homologues.

#### MOLLI60 [11]

This paper presents a general method for constructing matrices focused on a certain bias of amino acid composition. As initial data, proteins of bacteria belonging to the *Mollicutes* class were used, with genome biased towards A + T. Of the 14 bacterial genomes of the *Mycoplasma* species, by finding the greatest similarity (the standard method of bidirectional search BDBH for the best match was applied), 247 orthologous protein families were selected. To construct the matrix, the approach described in [2] was applied.

### Matrices based on the structural alignment

#### Johnson [12]

This matrix is based on the calculation of substitutions of amino acid residues in the structural alignments of 235 proteins from 65 homologous families. Most of the data used have a relatively low sequence identity of 15 to 40%.

#### Prlic [13]

Matrices were obtained on the basis of superposition of pairs of proteins having a similar structure, but low sequence identity, and are intended to compare evolutionarily distant proteins. Since structural alignments have several solutions, especially with low sequence identity, the following question arises: which of them is the most believable in terms of evolution? To build the matrices, alignments with the largest number of matching residues were used. Matrices obtained both on the basis of only homologous pairs and the entire set of structural alignments, including homologues and analogues, were considered.

#### Blake [14]

The study is based on two observations: data on structural superposition give a training sample to improve the alignment of distant homologs; and the main substitutable arrays of amino acid residues for distant homologs differ from those for closely related proteins. Based on the data of structural superpositions, a set of matrices of amino acid residue substitutions was constructed. These matrices use a known structural homology as the characteristics of the impact of evolution on the residue substitution profiles characterizing multiple alignment.

### Genetic code matrix

#### Benner [16]

The matrix was calculated on the assumption that for protein sequences spaced 1 PAM distance, the genetic code is the only factor leading to a drift in the amino acid composition. In other words, the influence of the code (i.e., the similarity of triplets) can occur either at the smallest evolutionary distances or in parts of the tertiary structure that have no effect on the function of the protein. To build the initial matrix, a database was used, including 1.7 million pairs of sequences. Further, the resulting matrix was extrapolated to a distance of 250 PAM by raising to the appropriate degree.

### Contacts energy matrix

#### Miyazawa [15]

The matrix is based on an estimation of the distribution of contacts in three-dimensional protein structures. The coordination number per residue is optimized in order to find the best fit between the observed and predicted partial energy. These new contact energies make it possible to improve their ability to discriminate the native structure from non-native folds in the dragging procedure.

## Supplementary information

**Additional file 1: Table S1.** Local alignment. Test sets: 30 PAM, 60 PAM, 120 PAM, Balibase.

**Additional file 2: Table S2.** Global alignment. Test sets: 30 PAM, 60 PAM, 120 PAM, Balibase.

**Additional file 3: Table S3.** Correlation of matrices.

## Data Availability

All matrices investigated in this article are available at https://drive.google.com/open?id=1g5pbaFwSftc-0VsC4kfjoNxEC-wTN8kx. The Random sequence generating tool used in this study is available at https://drive.google.com/drive/folders/1ECImZsgPDVg-8OpsIJUmVWOLKMvJbqEm. The datasets generated and analysed during the current study are available from the corresponding author on reasonable request.

## Abbreviations

PAM: Point Accepted Mutations
GOP: Gap Opening Penalty
GEP: Gap Extension Penalty
